# Karyotype diversity among predatory Reduviidae (Heteroptera)

**DOI:** 10.3897/CompCytogen.v8i4.8430

**Published:** 2014-12-18

**Authors:** Vanessa Bellini Bardella, Hélcio Reinaldo Gil-Santana, Francisco Panzera, André Luís Laforga Vanzela

**Affiliations:** 1 Departamento de Biologia, Instituto de Biociências, Letras e Ciências Exatas, IBILCE/UNESP, 15054-000, São José do Rio Preto, São Paulo, Brazil; 2 Laboratório de Diptera, Instituto Oswaldo Cruz, Rio de Janeiro, Rio de Janeiro, Brazil; 3 Sección Genética Evolutiva, Facultad de Ciencias, Universidad de la República, 11400 Montevideo, Uruguay; 4 Departamento de Biologia Geral, CCB, Universidade Estadual de Londrina, 86051-990, Londrina, Paraná, Brazil

**Keywords:** Cimicomorpha, DAPI/CMA_3_ banding, Heteroptera, holokinetic chromosomes, 18S rDNA

## Abstract

Species of infraorder Cimicomorpha of Heteroptera exhibit holokinetic chromosomes with inverted meiosis for sex chromosomes and high variation in chromosome number. The family Reduviidae, which belongs to this infraorder, is also recognized by high variability of heterochromatic bands and chromosome location of 18S rDNA loci. We studied here five species of Reduviidae (Harpactorinae) with predator habit, which are especially interesting because individuals are found solitary and dispersed in nature. These species showed striking variation in chromosome number (including sex chromosome systems), inter-chromosomal asymmetry, different number and chromosome location of 18S rDNA loci, dissimilar location and quantity of autosomal C-heterochromatin, and different types of repetitive DNA by fluorochrome banding, probably associated with occurrence of different chromosome rearrangements. Terminal chromosome location of C-heterochromatin seems to reinforce the model of equilocal dispersion of repetitive DNA families based in the “bouquet configuration”.

## Introduction

Species of the suborder Heteroptera share several cytogenetic features such as the occurrence of holokinetic chromosomes, inverted meiosis for sex chromosomes and variation in chromosome number (Ueshima 1979, Manna 1984, Pérez et al. 2000, Papeschi and Bressa 2006, Bardella et al. 2014a). Chromosome numbers vary from 2n = 4 in Nepomorpha to 2n = 80 in Cimicomorpha (Ueshima 1979, Manna 1984, Papeschi and Bressa 2006), and the latter infraorder displays the greatest karyotype diversity among the Heteroptera (Kuznetsova et al. 2011). These insects also exhibit diversity in heterochromatin distribution (Grozeva and Nokkala 2003, Grozeva et al. 2004, Ituarte and Papeschi 2004, Bressa et al. 2005, Franco et al. 2006, Panzera et al. 2010, Bressa et al. 2008, Chirino et al. 2013, Bardella et al. 2014a). Previous reports on C-heterochromatin in heteropterans showed that C-bands are terminally located. However, intercalary patterns are described in several species (Camacho et al. 1985, Dey and Wangdi 1990, Pérez et al. 1997, Papeschi et al. 2003, Ituarte and Papeschi 2004, Grozeva and Nokkala 2003, Angus et al. 2004, Grozeva et al. 2004, Waller and Angus 2005, Angus 2006, Bressa et al. 2008).

The 18S rDNA locus is the principal marker on chromosomes of Nepomorpha, Pentatomomorpha and Cimicomorpha (González-García et al. 1996, Papeschi et al. 2003, Cattani et al. 2004, Cattani and Papeschi 2004, Dias de Campos Severi-Aguiar and Azeredo-Oliveira 2005, Severi-Aguiar et al. 2006, Morielle-Souza and Azeredo-Oliveira 2007, Bressa et al. 2008, 2009, Grozeva et al. 2010, 2011, Poggio et al. 2011, 2013a, 2014, Panzera et al. 2012, Chirino et al. 2013a, Bardella et al. 2013). Of the 36 species of Pentatomomorpha studied until now, the rDNA loci are preferably located in autosomes with only four species with rDNA loci on the sex chromosomes (González-García et al. 1996, Bressa et al. 2009, Grozeva et al. 2011, Bardella et al. 2013). On the contrary, in Cimicomorpha, the location of rDNA loci are more heterogeneous: the hybridization sites are observed on autosomes, sex chromosomes or both simultaneously (Dias de Campos Severi-Aguiar and Azeredo-Oliveira 2005, Severi-Aguiar et al. 2006, Morielle-Souza and Azeredo-Oliveira 2007, Grozeva et al. 2010, 2011, 2013, 2014, Panzera et al. 2012, 2014, Poggio et al. 2011, 2013a, 2013b).

According to Schuh and Slater (1995), Cimicomorpha includes species with different habits, such as predatory and hematophagous (Reduviidae), phytophagous (Miridae) and ectoparasitic (Cimicidae and Polyctenidae). Predators are interesting because they act in the biological control of other insects, either in natural or agricultural environments (Schaefer and Panizzi 2000). The study of these insects is difficult because they are always found scattered in nature, without the formation of colonies. The small number of individuals obtained is a limiting factor for comparative analyses of relatedness and karyotype evolution, as well as for population approaches. We made great efforts to obtain a large number of predators of Cimicomorpha to increase our knowledge of the karyotypical structure of these insects. Our goal was to generate a good volume of data and to compare them with the results previously reported for other heteropteran groups. The results presented here for the family Reduviidae provide information on karyotype organization, including the distribution of heterochromatin and location of 18S rDNA sites. These analyses reinforce the model of equilocal dispersion of repetitive DNA families based in the “bouquet organization”.

## Materials and methods

**Figure 1.**
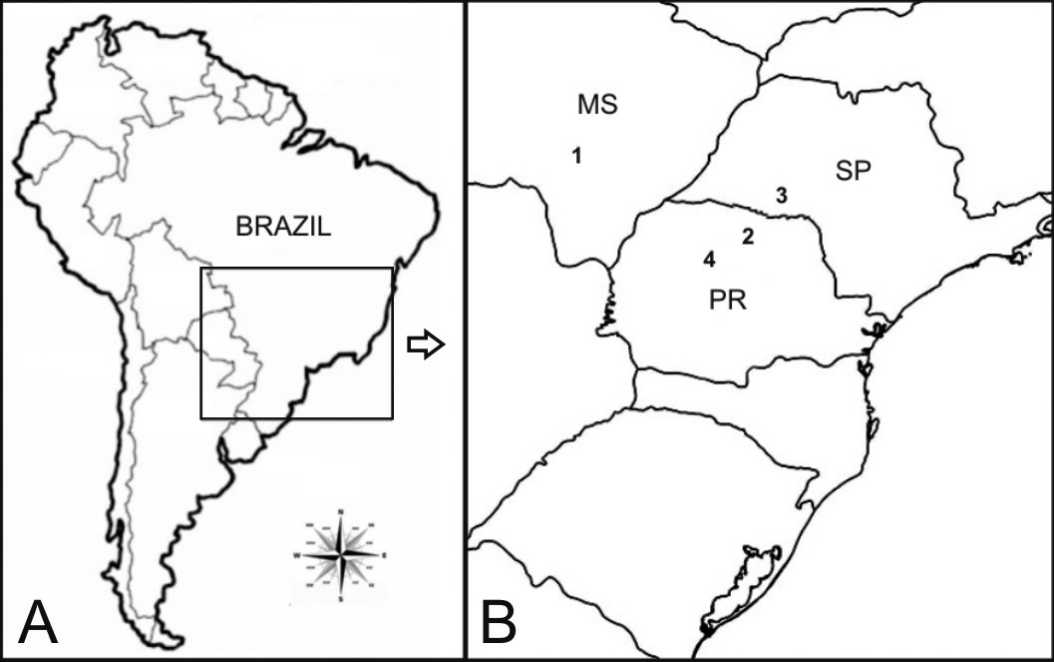
Maps of South America and Brazil (**A**). The section in **B** indicates the position of the states with collection points (SP: São Paulo, MS: Mato Grosso do Sul and PR: Paraná). The locations 1, 2, 3 and 4, which indicate the cities where heteropterans were collected, are specified in Table 1.

Five species of Heteroptera belonging to the family Reduviidae (subfamily Harpactorinae) were collected in the South and Southeast regions of Brazil, and information about the collection localities is given in Fig. 1 and Table 1. Conventional karyotypes of *Apiomerus lanipes* (Fabricius, 1803) and *Cosmoclopius nigroannulatus* (Stål, 1860) were previously described (Poggio et al. 2007), while all cytogenetic information on *Zelus laticornis* (Herrich-Schäffer, 1853), *Montina confusa* (Stål, 1859) and *Repipta flavicans* (Amyot & Serville, 1843) is new. Gonads were dissected out and the seminiferous tubules were ﬁxed in a solution of methanol-acetic acid (3:1, v:v) and stored at 20°C below zero. For the preparation of slides, tubules were incubated in 45% acetic acid for 10 min at room temperature, and squashed in a drop of 45% acetic acid. Coverslips were removed after freezing in liquid nitrogen, and the slides air-dried. For conventional staining the slides were treated with 1N HCl for 6 min at room temperature and stained with 2% Giemsa for 1 min at room temperature. The samples were air-dried and mounted with Entellan. Chromosome measurements were made in ﬁve metaphases I, with similar chromosome condensation, for each species. The measurement was performed manually, using a needle point compass. Chromosome pairs were arranged in decreasing size, according to the average size and standard deviation. The sex chromosomes were distinguished by the characteristic arrangement in metaphase I and were measured separately since they exhibit univalent behavior.

For chromosome C-banding (Sumner 1982, with modifications), slides were aged for three days after removal of coverslips. Afterwards, the slides were incubated in 0.2 N HCl for 10 min at room temperature, 5% barium hydroxide at 60°C for 2 min, and 2× SSC, pH 7.0, at 60°C for 60 min. Samples were treated with 30 µl of each ﬂuorochrome: 0.5 mg/ml chromomycin A_3_ (CMA_3_/Sigma) for 1.5 h at room temperature and 2 μg/ml 4’6-diamidino-2-phenylindole (DAPI/Invitrogen) for 30 min at room temperature. Preparations were mounted with a medium composed of glycerol/McIlvaine buffer, pH 7.0 (1:1, v:v), plus 2.5 mM MgCl_2_.

Fluorescent *in situ* hybridization (FISH) was done as described in Bardella et al. (2010) and performed on samples of at least two individuals per species. The p*At* 05 clone, containing a partial sequence of the 18S rDNA of *Antiteuchus tripterus* (Fabricius, 1787) (Pentatomidae, Pentatomomorpha), was labeled with digoxigenin-11-dUTP by nick translation (DIG-nick translation mix Roche prepared according to the procedures recommended by the manufacturer). Preparations were treated with 30 µl of hybridization mixture containing 4 µl of labeled probe (100 ng), 15 µl of 100% formamide, 6 µl of 50% polyethylene glycol, 3 µl of 20×SSC, 1 µl of 10% SDS and 1 µl of water. Chromosome denaturation/renaturation was done at 90°C for 10 min using a thermal cycler, and hybridization was performed for 12 h at 37°C in a humidified chamber. Post-hybridization washes were carried out at different concentrations of SSC buffer (3.17M NaCl and 0.34M Na_3_C_6_H_5_O_7_), with 60% stringency due to heterologous hybridization. For detection, anti-digoxigenin-rhodamine in 5% BSA/4× SSC/0.2% Tween 20 (1:100, v:v) was used. The post-detection washes were performed in 4× SSC/0.2% Tween 20 at room temperature. Slides were mounted with 26 µl of DABCO solution (1,4-diaza-bicyclo (2.2.2)-octane (2.3%), 20 mM Tris-HCl, pH 8.0, (2%) and glycerol (90%) in distilled water), 2 µl of 2 µg/ml DAPI and 1 µl of 50 mM MgCl_2_.

All chromosome images were acquired separately in grayscale mode using a Leica DM 4500 B epifluorescence microscope equipped with a very high sensitivity, 1.4 MPixel resolution, firewire interface Leica DFC300 FX camera. Pseudo coloration of blue/red colors for DAPI, greenish for CMA_3_ and greenish-yellow for rhodamine were done using Leica IM50 4.0 software, as well as the overlapping of images.

**Table 1.** Information about predatory Reduviidae predators. The numbers before the city names indicate the position on the map (Fig. 1) and capital letters refer to the Brazilian states: SP: São Paulo, MS: Mato Grosso do Sul and PR: Paraná. The average sizes of chromosomes of all species are presented in µm. with standard deviation. Asterisk indicates the size of sex chromosomes, CN = chromosome number, CP = chromosome pairs (univalent for sex chromosomes), SC = sex chromosome, LSC = large sex chromosome, SSC = small sex chromosome and FSC = fragmented sex chromosome.

Species	*Apiomerus lanipes*		*Cosmoclopius nigroannulatus*		*Zelus laticornis*		*Montina confusa*		*Repipta flavicans*	
Number of Males	6		4		5		3		5	
Localities	(1) Nova Alvorada do Sul-MS		(2) Londrina-PR		(3) Assis-SP		(3) Assis-SP		(4) Borrazópolis-PR	
Coordinates	21°23.058'S, 54°23.012'W		23°18.394'S, 51°12.139'W		22°28.645'S, 50°20.983'W		22°28.645'S, 50°20.983'W		23°56.225'S, 51°35.280'W	
CN	2n = 22+XY		2n = 24+X_1_X_2_X_3_Y		2n = 24+XY		2n = 12+XY		2n = 18+XY	
CP	2n	n	2n	n	2n	n	2n	n	2n	n
1	4.64 ± 0.33	2.32	2.89 ± 0.27	1.45	3.79 ± 0.62	1.90	4.95 ± 0.64	2.48	3.83 ± 0.45	1.92
2	3.76 ± 0.29	1.88	2.74 ± 0.33	1.37	3.13 ± 0.40	1.57	4.83 ± 0.53	2.42	3.09 ± 0.28	1.55
3	3.50 ± 0.27	1.75	2.58 ± 0.11	1.29	2.99 ± 0.48	1.50	4.10 ± 0.35	2.05	2.96 ± 0.17	1.48
4	3.50 ± 0.27	1.75	2.50 ± 0.00	1.25	2.88 ± 0.52	1.44	2.63 ± 0.47	1.32	2.79 ± 0.17	1.40
5	3.40 ± 0.22	1.70	2.26 ± 0.13	1.13	2.70 ± 0.35	1.35	2.63 ± 0.47	1.32	2.65 ± 0.10	1.33
6	3.30 ± 0.00	1.67	2.20 ± 0.00	1.10	2.54 ± 0.09	1.27	2.56 ± 0.35	1.28	2.60 ± 0.12	1.30
7	3.30 ± 0.00	1.65	2.14 ± 0.13	1.07	2.54 ± 0.09	1.27	2.05 ± 0.31*	1.02^ LSC^	2.48 ± 0.21	1.24
8	3.25 ± 0.11	1.65	2.14 ± 0.13	1.07	2.42 ± 0.22	1.21	1.95 ± 0.31*	0.97^ SSC^	2.40 ± 0.24	1.20
9	3.08 ± 0.25	1.63	1.96 ± 0.25	0.98	2.36 ± 0.23	1.18	-		2.33 ± 0.35	1.17
10	2.96 ± 0.26	1.54	1.96 ± 0.25	0.98	2.26 ± 0.25	1.13	-		1.80 ± 0.33*	0.9^ LSC^
11	2.64 ± 0.43	1.48	1.78 ±0.16	0.89	2.20 ± 0.21	1.10	-		1.75 ± 0.33*	0.87^ SSC^
12	3.35 ± 0.17*	1.68^SC^	1.60 ± 0.00	0.80	2.08 ± 0.34	1.04	-		-	
13	3.35 ± 0.17*	1.68^SC^	1.48 ± 0.18*	0.74^ LSC^	1.56 ± 0.58*	0.78^ LSC^	-		-	
14			0.60 ± 0.18*	0.30^FSC^	1.32 ± 0.58*	0.66^ SSC^				
15			0.40 ± 0.18*	0.20^ FSC^						
16			0.38 ± 0.18*	0.19^ FSC^						

## Results

**Figure 2.**
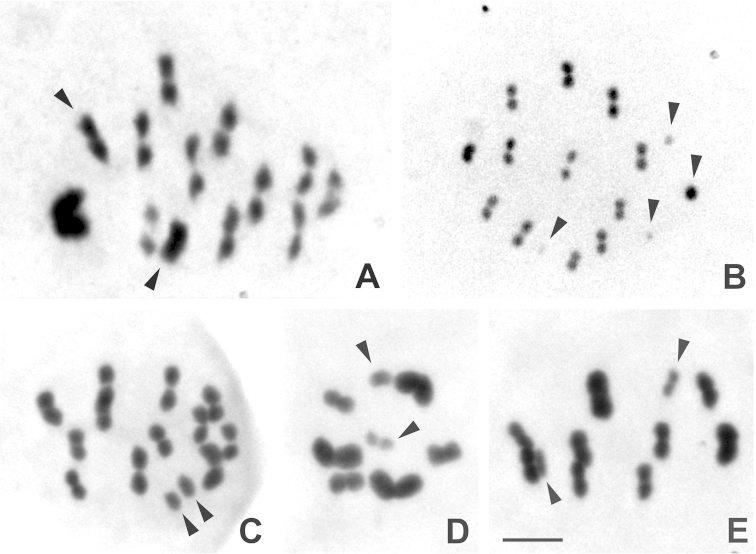
Conventional staining with 2% Giemsa of meiotic chromosomes of five species of Reduviidae. **A** *Apiomerus lanipes*: Metaphase I. 2n = 22 + XY **B** *Cosmoclopius nigroannulatus*. Metaphase I. 2n = 24 + X_1_X_2_X_3_Y **C** *Zelus laticornis* Metaphase I. 2n = 24 + XY **D** *Montina confusa*. Metaphase II. 2n = 12 + XY **E** *Repipta flavicans*. Metaphase I. 2n = 18 + XY. The arrowheads indicate the sex chromosomes. Bar = 5µm.

**Figure 3.**
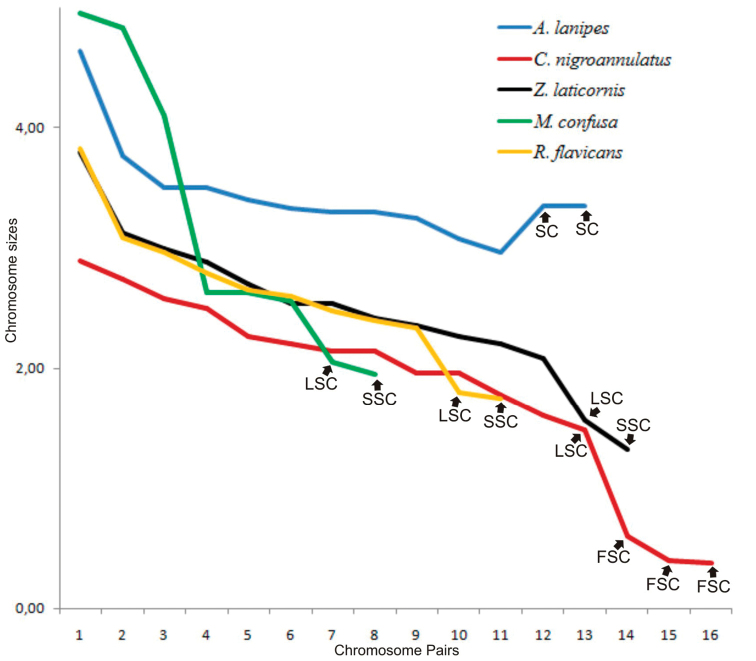
Graph showing the difference in karyotype in five species of Reduviidae, based on the decrease in chromosome size. SC indicates the position of the sex chromosomes of similar size, LSC points to the large sex chromosome, SSC shows the small sex chromosome, and FSC indicates the fragmented sex chromosomes. Note that *Montina confusa* displays the karyotype with a great sized variation among the five species analyzed, and *Apiomerus lanipes* is the only species with sex chromosomes of intermediate size relative to the autosomes.

The chromosome numbers found for the five species of Reduviidae were 2n = 22 + XY in *Apiomerus lanipes*, 2n = 24 + X_1_X_2_X_3_Y in *Cosmoclopius nigroannulatus*, 2n = 24 + XY in *Zelus laticornis*, 2n = 12 + XY in *Montina confusa* and 2n = 18 + XY in *Repipta flavicans* (Fig. 2A–E, respectively). In *Zelus laticornis* and *Repipta flavicans* the size of chromosomes decreased gradually (Fig. 2 and Fig. 3). In *Apiomerus lanipes*, chromosome asymmetry was due to the existence of a larger autosomal pair. *Cosmoclopius nigroannulatus* exhibited three sex chromosomes (X) with reduced size, and *Montina confusa* showed three larger autosomal pairs (Fig. 3). In all species, the sex chromosomes were smaller of the chromosome complement; except in *Apiomerus lanipes*, where the sex chromosomes exhibited intermediate relative sizes (Fig. 3).

**Figure 4.**
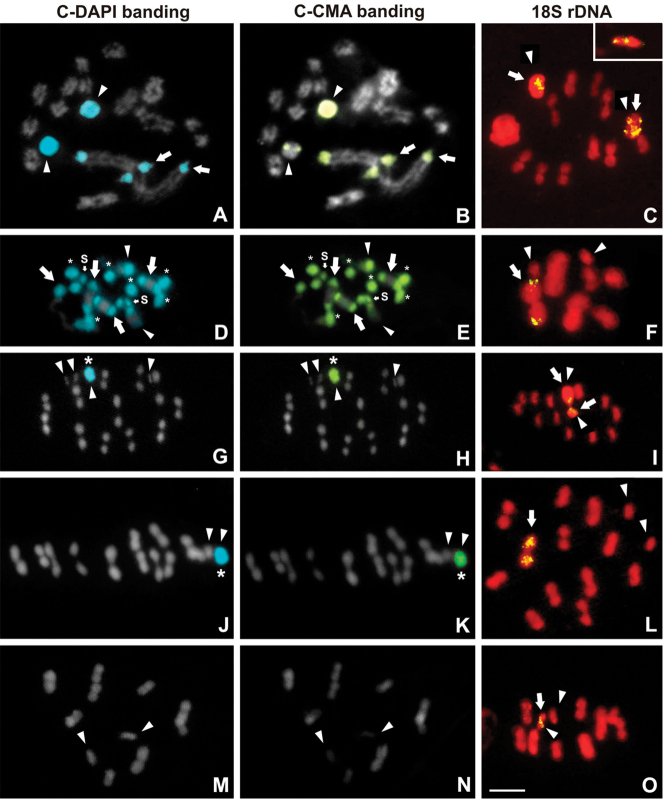
C-DAPI/CMA banding and FISH with 18S rDNA probe in five predatory species of Reduviidae. **A–C** *Apiomerus lanipes*. Diplotene: terminal DAPI^+^/CMA ^+^ bands in the largest bivalent (arrows), one sex chromosomes totally DAPI^+^/CMA^+^ (arrowhead) and the other sex chromosome totally DAPI^+^ (arrowhead) with a terminal CMA^+^ dot. In metaphase I, the hybridization rDNA signals are located at terminal positions of both sex chromosomes (arrow and box - metaphase II) **D–F** *Montina confusa*. Gonial mitosis with two autosomal pairs and both sex chromosomes exhibiting DAPI^+^/CMA^+^ bands at both terminal regions (arrow and the word S, respectively), one autosomal pair with DAPI^+^/CMA^+^ band at one terminal region (arrowhead) and three autosomal pairs totally DAPI^+^/CMA^+^(asterisk). In metaphase **I**, ribosomal loci are located on the largest bivalent (arrow) **G–I** *Cosmoclopius nigroannulatus*. In metaphase I, the Y chromosome appear entirely DAPI^+^/CMA^+^ (asterisk) and hybridization signals of rDNA on two sex chromosomes in metaphase II (arrows). Note the aggregation of the three X chromosomes **J–L** *Zelus laticornis*. Metaphase I has one sex chromosome totally DAPI^+^/CMA^+^ (asterisk), and the rDNA sites are situated on one bivalent (arrow) **M–O** *Repipta flavicans*. Diakinesis without heterochromatic regions. The hybridization signals are located on one sex chromosome in metaphase I (arrow). Arrowheads indicate the sex chromosomes. Bar = 5µm.

The predominant sex determination system was the simple XY in the species studied, except *Cosmoclopius nigroannulatus*, which displayed X_1_X_2_X_3_Y (Fig. 2B). The difficulty of keeping these species in captivity made it impossible to obtain eggs, and this prevented the differentiation of the sex chromosomes X and Y for the species with a simple sex chromosome system. Therefore, these chromosomes are named here generically as only “sex chromosomes”. The comparison of measurements of sex chromosomes showed that X_1_X_2_X_3_ of *Cosmoclopius nigroannulatus* were five times smaller than the sex chromosomes of *M. confusa, R. flavicans* and *Zelus laticornis*, and up to ten times smaller than the sex chromosomes of *Apiomerus lanipes* (Table 1 and Figs 2–4).

Fluorescent C-chromosome banding exhibited a large variability in the occurrence of C-DAPI^+^/CMA_3_^+^ bands among the five species:

*Apiomerus lanipes*: Only the largest autosomal pair showed terminal C-DAPI^+^/CMA_3_^+^ bands (Fig. 4A–B). The heterochromatic sex chromosomes of this species exhibit different fluorescent patterns (Fig. 4A–B). One sex chromosome appeared totally C-DAPI^+^/CMA_3_^+^, and the other was totally C-DAPI^+^ with C-CMA_3_^+^ band observed as subterminal dots (arrowheads in the Fig. 4A–B).

*Montina confusa*: A large number of heterochromatic bands is observed: the two largest autosomes and both sex chromosomes exhibited C-DAPI^+^/CMA_3_^+^ bands in both terminal regions. The third autosomal pair showed a C-DAPI^+^/CMA_3_^+^ band in only one terminal region, whereas the three smaller pairs were totally C-DAPI^+^/CMA_3_^+^ (Fig. 4D–E).

*Cosmoclopius nigroannulatus*: Autosomal complement not exhibit fluorescence banding. The Y chromosome is totally C-DAPI^+^/CMA_3_^+^ (Fig. 4G–H),

*Zelus laticornis*: only one sex chromosome was totally C-DAPI^+^/CMA_3_^+^ (Fig. 4J–K).

*Repipta flavicans* exhibited no fluorescent bands in autosomes and sex chromosomes (Fig. 4M–N).

FISH experiments with the 18S rDNA probe showed variation in number, location, and signal intensity. In all species the hybridization signals always appeared at terminal chromosome positions. In *Apiomerus lanipes*, both sex chromosomes showed hybridization signals (Fig. 4C). In *Cosmoclopius nigroannulatus*, one of the signals of 18S rDNA was located on the largest sex chromosome (Y), whereas the other ribosomal signal was observed on one of the fragmented X chromosomes (Fig. 4I), which had a CMA_3_-negative signal after C-CMA banding (Fig. 4H). In *Repipta flavicans*, a hybridization signal was observed on one sex chromosome (Fig. 4O). In *Montina confusa* (Fig. 4F) and *Zelus laticornis* (Fig. 4L), hybridization signals were observed on a large autosomal bivalent.

## Discussion

Species of Reduviidae show low variation in chromosome number, from 2n = 12 in the genus *Polididus* Stål, 1858 (Manna and Deb-Mallich 1981) up to 2n = 34 in the genus *Bagauda* Bergroth, 1903 (Ueshima 1979), when compared with other families of Cimicomorpha, such as Miridae (2n = 14 to 80) and Cimicidae (2n = 14 to 50) (Kuznetsova et al. 2011). Many of these chromosome variations have been associated with chromosomal rearrangements such as fusion and fragmentation (Ueshima 1979, Papeschi and Bidau 1985, Papeschi 1988, 1994, Rebagliati et al. 2001, Bressa et al. 2002, Papeschi and Bressa 2006, Poggio et al. 2007, 2009, 2014, Grozeva et al. 2010, Chirino et al. 2013, Chirino and Bressa 2014). Although these arguments have been proposed considering the occurrence of rearrangements, there is not much evidence of these changes in Heteroptera. Rearrangements are more precisely evidenced when trivalents, multivalents or robust cytogenetic markers (heterochromatin, rDNA sites or others) are noted. Samples of these events were reported for species of *Belostoma* Latreille, 1807 (Papeschi 1994, 1996) and *Triatoma infestans* (Klug, 1834) (Poggio et al. 2013b). Other examples of chromosome changes were reported in insects of the family Aradidae, where fusions were important for karyotype evolution (Jacobs 2003), as well as the dysploidy that originated the neoXneoY sex system in *Dysdercus albofasciatus* Guérin Meneville, 1831 (Bressa et al. 2009). Dysploidy is recognized as an important evolutionary mechanism for karyotype differentiation in organisms with holokinetic chromosomes, for both plants (Guerra 2008) and animals (Bardella et al. 2014a). Due to the lack of phylogenetic analyses as well as the absence of chromosome markers for most heteropterans, the evolutionary direction for certain rearrangements is very speculative, especially in heteropteran predators. However, there are sporadic examples where chromosome rearrangements can be supposed, as observed in *Cosmoclopius nigroannulatus*, where numerical diversity is clearly linked to the fragmentation of sex chromosomes (Papeschi 1994, 1996, Poggio et al. 2007, 2013a, 2014).

Of the five karyotypes of Reduviidae studied here, two (*Repipta flavicans* and *Zelus laticornis*) showed a gradual decrease in size. This feature is common in Heteroptera, and it has been observed in species of different families, such as *Holhymenia rubiginosa* Breddin, 1904, Coreidae (Bressa et al. 2008) and *Edessa rufomarginata* (De Geer, 1773), Pentatomidae (Rebagliati et al. 2003). On the other hand, the substantial dissimilarities in the autosomal size or between sex chromosomes and autosomes were marked in three of the species here analyzed. In *Apiomerus lanipes*, the presence of a greater bivalent could be associated with a reduction in their chromosome number (2n = 24), when compared with the modal number of the subfamily Harpactorinae, 2n = 26 (Poggio et al. 2007). A similar situation was observed in *Dichelops furcatus* (Fabricius, 1775), (Rebagliati et al. 2001), and in *Lygaeus alboornatus* Blanchard, 1852 (Bressa et al. 2002), in which a very large bivalent probably originated from a chromosome fusion. In *Cosmoclopius nigroannulatus*, as discussed above, the reduced size of three X chromosomes is due to fragmentation events, as reported by Poggio et al. (2007). The most striking case found here was the karyotype of *Montina confusa*. Grozeva et al. (2006) reported more than one large chromosome in *Macrolophus costalis* Fieber, 1858 (Miridae). In heteropterans, significant variation in karyotype size may be associated not only with chromosomal rearrangements, but also with differential accumulation of heterochromatin, able to change the set size (Panzera et al. 1995, 2004, Bressa et al. 2008, Chirino et al. 2013, Bardella et al. 2014b). However, this does not seem be the case for *Apiomerus lanipes* and *Montina confusa*, because if we disregard the heterochromatin, these chromosomes are still very large.

The variation in the content and distribution of heterochromatin in autosomes and sex chromosomes is well documented in heteropteran species, and occurs mainly in the terminal chromosomal regions (Grozeva and Nokkala 2002, Bressa et al. 2005, Panzera et al. 2010, Grozeva et al. 2010, Chirino et al. 2013, Suman and Kaur 2013, Poggio et al. 2014, Bardella et al. 2014a). This common feature was observed only in *Montina confusa* among the predator species studied here. On the other hand, *Cosmoclopius cosmoclopius* and *Zelus laticornis* showed heterochromatin located only in one of the sex chromosomes. The heterochromatic profile reported in *Apiomerus lanipes* is similar to that observed for *Triatoma infestans*, but the latter displays a greater number of bivalents with terminal heterochromatic regions (Panzera et al. 1995, 2010, Bardella et al. 2014b). *Triatoma infestans* was the best studied species of Reduviidae in relation to the distribution of heterochromatin. This species exhibits bands in terminal chromosome regions, but there is a variation in the chromosome pairs carrying bands, which is associated with the geographic distribution of each population in South America (Panzera et al. 1992, 1995, 2004, 2014). High interspecific variation in distribution of heterochromatin has also been reported for other species of Cimicomorpha (Grozeva and Nokkala 2001, Panzera et al. 2010) and Pentatomomorpha (Bardella et al. 2014a). Despite the high variability found in the content and distribution of heterochromatin, the constancy in the positioning of bands in terminal chromosome regions suggests that mechanisms of dispersion of heterochromatin could be associated with positioning of satDNA in interphase. The model of “bouquet polarization,” which postulates that chromosomes can be closely associated with the nuclear envelope through their ends, could support the idea of the sharing of repetitive DNA families at terminal chromosomal regions. The “bouquet polarization” model was proposed by Rodriguez Iñigo et al. (1996) when cells in the transition interphase-prophase I of *Dociostaurus genei* (Ocskay, 1832) (Orthoptera) were studied. Among Heteroptera, the “bouquet” has been mentioned for *Pyrrhocoris apterus* Linnaeus, 1758. (Suja et al. 2000) and *Graphosoma italicum* (O.F. Muller, 1766) (Vieira et al. 2009). Except for *Holhymenia histrio, H. rubiginosa, Macrolophus costalis* and *Spartoceras batatas* (Fabricius, 1758), which show interstitial bands on some chromosomes (Franco et al. 2006, Grozeva et al. 2006, Bressa et al. 2008, Bardella et al. 2014a), the terminal pattern of heterochromatin distribution, such as that found here in *Montina confusa*, was also found in almost all species of Heteroptera. The total absence of bands, as found here in *Repipta flavicans*, has been seen in different families of Heteroptera: Reduviidae (Poggio et al. 2011); Belostomatidae (Papeschi and Bidau 1985, Papeschi 1994, Papeschi and Bressa 2006), Coreidae (Bressa et al. 2005, Bardella et al. 2014a), Pentatomidae and Pyrrhocoridae (Bressa et al. 2009, Bardella et al. 2014a). This suggests that the presence or not of heterochromatin may be intrinsic in each genome, regardless of the phylogenetic relationships of the species studied to date.

In heteropteran species, many C-heterochromatic bands can be AT or GC-rich, such as in *Montina confusa* (Rebagliati et al. 2003, Bressa et al. 2005, Franco et al. 2006, Bardella et al. 2010, 2012, 2014a, Chirino et al. 2013). In this way, the example of *Triatoma infestans* can be highlighted because the distinct repetitive DNA families (AT- and GC-rich) appear adjacently arranged at terminal chromosome regions (Bardella et al. 2014b). On the other hand, species with small amounts of constitutive heterochromatin generally exhibit only CG-rich bands or dots associated with the nucleolar organizer regions (NORs), as observed in *Graphosoma italicum* (González-García et al. 1996), among others (Cattani et al. 2004, Papeschi and Bressa 2006, Bardella et al. 2010, Grozeva et al. 2013, Chirino et al. 2013). Only in few species, NORs associated with AT-rich regions have been observed (Fossey and Liebenberg 1995, Bardella et al. 2010). Differently, *Zelus laticornis* showed CG and AT-rich heterochromatin completely restricted to only one of the sex chromosomes without association with the NORs. Similar cases have been reported in *Triatoma brasiliensis* Neiva, 1911 and *Triatoma rubrovaria* Blanchard, 1834 (Bardella et al. 2010).

The FISH studies in five species of predators studied here showed a variation in number (1-3) and distribution (autosomes and/or sex chromosomes) of 18S rDNA sites. These variations are included within the range previously reported for Reduviidae (Bardella et al. 2010, Panzera et al. 2012). For this group, Poggio et al. (2011) suggested that the 18S rDNA sites are generally located at the terminal position on the X chromosome, or on both sex chromosomes in species with simple sex chromosome system (XY). However, in most cases the ribosomal loci are located at terminal position on an autosomal pair in species with multiple sex chromosomes (X_n_Y). However, our data on *Cosmoclopius nigroannulatus*, which shows fragmentation of the X chromosome, suggests an additional situation for the distribution of 18S rDNA sites, since the rDNA signals appeared on both one of the fragmented X chromosomes and Y chromosome. The presence of 18S or 45S rDNA loci in one or more sex chromosomes has also been observed in several reduviid species from the subfamilies Triatominae (Severi-Aguiar et al. 2006, Panzera et al. 2012) and Reduviinae (Poggio et al. 2013a) with multiple sex chromosome system. There is at least one example, *Dysdercus albofasciatus*, where the original X chromosome was inserted into the NOR-autosome next to the rDNA cluster in an ancestor carrying the X0 system, resulting in a neo-sex-chromosome system (Bressa et al. 2009). We did not observe chromosomal rearrangements associated directly with the mobility of 18S rDNA sites in the reduviids. However, the variation in the chromosomal location of rDNA loci seems to be more common in reduviids from the Cimicomorpha infraorder than in the Pentatomomorpha infraorder (Panzera et al. 2012, Bardella et al. 2013, Poggio et al. 2013a). This variability indicates different evolutionary pressures for the 18S rDNA distribution in the suborder Heteroptera, as in other insect groups (Nguyen et al. 2010).

Despite the five analyzed species belong to the same subfamily (Harpactorinae) and share the predatory habit (Zhang and Weirauch 2013), we observe different evolutionary pathways in their chromosomes based on the extensive cytogenetic differences: i) great variation in chromosome number, ii) inter-chromosomal asymmetry, iii) simple and multiple sex systems, iv) different number and chromosome location of 18S rDNA loci, v) dissimilar location and quantity of autosomal C-heterochromatin, and vi) different types of repetitive DNA by fluorochrome banding. The chromosome diversity found in this study clearly shows the need for analysis of a large number of species to establish evolutionary patterns in predator reduviids.
